# Expectantly waiting: a survey of thyroid surgery wait times among Canadian Otolaryngologists

**DOI:** 10.1186/1916-0216-42-47

**Published:** 2013-09-11

**Authors:** Maria Brake, Paige Moore, S Mark Taylor, Jonathan Trites, Scott Murray, Rob Hart

**Affiliations:** 1 Division of Otolaryngology-Head and Neck Surgery, Dalhousie University, Halifax, NS, Canada; 2 Room 826, Victoria Building, QEII Health Sciences Centre, 1278 Tower Road, Halifax, NS B3H-2Y9, Canada

**Keywords:** Thyroid nodule, Thyroid cancer, Wait times, Thyroidectomy, Otolaryngology referral

## Abstract

**Background:**

Our objective is to highlight discrepancies between actual wait times and perceived appropriate wait times for various thyroid pathologies among Otolaryngology- Head and Neck Surgeons in Canada; and to identify specific diagnoses/pathologies where wait times could be improved.

**Methods:**

A questionnaire was distributed to all practicing CSO-HNS members. Questions focused on actual wait times for initial consults and surgery within individual practices, in the setting of various thyroid pathologies. Respondents were also asked to state wait times that they felt were appropriate for each scenario. Wilcoxon signed-rank tests were performed to determine statistically significant differences between actual and appropriate wait times.

**Results:**

For most scenarios, the actual wait times were significantly longer than most physicians felt were appropriate; these scenarios included time to initial consult for undiagnosed nodules, time to surgery for confirmed malignancies, and time to completion thyroidectomy for surgically confirmed malignancies.

**Conclusions:**

Wait times for thyroid consults and surgeries in Canada are longer than physicians feel are appropriate. The authors hope that this survey may spur a move towards a national consensus on appropriate wait times for the treatment of thyroid pathology.

## Introduction

Wait times for specialized medical services is a recurring issue in publicly funded healthcare systems such as Canada’s, where the reduction of wait times remains an ongoing priority in the government’s healthcare agenda [1]. The Canadian federal and provincial governments put forth a joint accord in 2004 called *The 10-year plan to strengthen healthcare*[2], which aimed to address many problem areas including wait times [3]. The accord dedicated $5.5 billion to a wait-times reduction fund, designed to help provincial and territorial governments reduce wait times in five priority areas: cancer care, cardiac care, diagnostic imaging, joint replacement, and sight restoration [4]. While recent federal reviews on the accord’s progress indicate that benchmarks have been successfully established for these areas, the cancer care priority area only addresses radiation therapy benchmarks, omitting surgical and other oncology treatments [5,6]. Physicians and government bodies alike are calling for participation from all specialty areas in order to address wait time benchmarks more comprehensively [6].

There currently exists no suggested wait times specific to thyroid surgery. Thyroid cancer has doubled in incidence in much of the developed world over the past few decades [7]. It’s incidence is increasing more rapidly than any other cancer in Canada; with a 6.9% increase per year since 2002 in women, and 6.8% increase per year since 1998 in men [8]. It is believed that this rise in incidence may be attributable to more organic factors than simply advances in diagnostic techniques [7]. Despite thyroid cancer being among the least aggressive oncologic diagnoses, and a lack of evidence that prolonged wait times to treatment are associated with poorer outcomes, these patients experience as much or more psychological distress while waiting for treatment as other cancer populations [9,10]. This distress can also continue for years after long-term remission [11]. It is therefore important to determine if these patients are waiting inappropriate lengths of time for their procedures to eliminate undue anxiety to the patient, and to contribute to our country’s ongoing efforts to reduce wait times.

To date, there are few studies examining wait times in Otolaryngology [12], and even fewer looking at wait times for specific diagnoses or procedures. This study aims to depict current wait times in Canada for thyroid consults and surgeries given various pathologies, to determine what Canadian otolaryngology thyroid surgeons feel are appropriate wait times for these procedures, and whether there is a statistically significant difference between the two. There have been no prior publications addressing thyroid surgery wait times at the time of publication.

## Materials and methods

### Survey development

Opinion online survey software was used to develop a questionnaire incorporating questions regarding physician demographics and thyroid wait time data. The survey consisted of 29 questions: 3 inclusion/exclusion questions; 6 physician demographic questions; and a series of 10 pairs of questions regarding actual and expected wait times for various thyroid diagnoses/clinical scenarios. The clinical scenarios consisted of 2 “time to initial consult” questions, and 8 “time to OR” questions, and were worded as such:

1. a) How long do newly consulted patients with undiagnosed nodules (>1 cm) wait before being seen for assessment?

OR

a. How long do patients in your practice wait for surgery if FNA shows, or is suspicious for, papillary carcinoma?

b. How long do you feel is the appropriate wait time for the patients mentioned in a)?

Respondents estimated the average wait time their patients experience for a given scenario (part a), and also reported the wait time they felt is appropriate for the same scenario (part b). Responses were multiple choice, and consisted of multiple wait time intervals (ie, 0–4 weeks; 4–8 weeks; 8–12 weeks; 3–6 months, > 6 months). The intervals were adjusted for certain questions to ensure accurate data capture.

Exclusion questions at the beginning of the survey ensured participants were actively practicing Otolaryngologists in Canada, registered with the Canadian Society of Otolaryngology-Head and Neck Surgery (CSO-HNS), who assess *and* operate on patients presenting with thyroid pathology. Questions were set such that residents, fellows, emeritus, international, and non-practicing CSO-HNS members were excluded from the survey responses; as were members who do not assess or operate on thyroid patients.

The physician demographic variables recorded included: province of practice; type of practice (academic, community, or both); age; years in practice; fellowship (if yes, whether it was in head and neck oncology); and number of new thyroid patient consults per month.

### Survey distribution

An email was sent to all registered CSO-HNS members via a central distribution address and requested participation from members who performed thyroid surgery. The emails containing notification of the survey included a weblink to the survey. Email notices were sent three times at monthly intervals. The email was sent to a total of 465 CSO-HNS members.

### Statistical analysis

Survey data were analyzed using the Statistical Package for the Social Sciences, version 11.5 (SPSS, Chicago, IL). Wilcoxon signed-rank tests were used to identify differenced between the *actual* and *accepted* wait times. Level of significance was set at α = 0.05 (with Bonferroni correction α was adjusted to 0.005). Linear regression models were used to evaluate demographic factors that significantly influenced the perceived appropriate wait times with α = 0.05.

## Results

There were a total of 92 responses, and 75 met the inclusion criteria. Of the respondents, 45% represented solely academic practitioners, 35% represented solely community practitioners, and 20% practiced in both environments. Fifty-seven percent of respondents were fellowship-trained, only 46% of these being specifically in head and neck oncology.

The median range of participant ages were 41–50 years, with 45% of participants aged 40 years or younger and 55% of participants greater than 40 yrs. Fifty-one percent of participants had been practicing 10 years or less, with the median range being 5 – 10 years.

The results of the non-parametric paired difference testing can be found in Table 1. All results were statistically significant (p < 0.005) except for three scenarios: time to consults for both undiagnosed and diagnosed thyroid nodules, as well as time to surgery for a diagnosis of medullary thyroid cancer (MTC). Table 1 plots the median wait times in each scenario; whereas our statistical analysis was performed using paired differences (Wilcoxon signed rank test). It is therefore possible to have a significant difference between actual and expected wait times even when the median values are the same.

**Table 1:** Results of statistical analysis of actual and appropriate wait times for various thyroid pathologies in Canada

**Clinical scenario**	**Median wait times**			
	**Actual**	**Appropriate**	**Mean paired differences**^**†**^	**p-value**^**††**^
**Time to see new consults with:**				
Undiagnosed nodule >1 cm	4-8 weeks	4-8 weeks	0.57	0.0351
Positive/suspicious FNA	2-4 weeks	2-4 weeks	0.25	0.0351
**Time to OR if FNA shows:**				
Papillary Carcinoma	6-8 weeks	4-6 weeks	0.61	**0.0002**
Follicular neoplasm	8-12 weeks	8-12 weeks	0.57	**0.0002**
AUS*	8-12 weeks	6-8 weeks	0.51	**0.0013**
Indeterminate/Non-diagnostic	3-6 months	2-3 months	0.52	**0.0002**
Medullary carcinoma	2-4 weeks	2-4 weeks	0.34	0.0198
Benign symptomatic disease	3-6 months	3-6 months	0.61	**0.0001**
**Time to completion thyroidectomy if initial hemi shows:**				
Papillary or Follicular Ca	6-8 weeks	4-6 weeks	0.52	**0.0041**
Medullary Ca	4-6 weeks	2-4 weeks	0.29	**0.0027**

^†^*As determined by Wilcoxon signed-rank test;*^††^α=0.005, *significant results in bold*; *Atypical cells of undetermined significance.

The linear regression model revealed only a few statistical significant relationships. There were significant negative correlations between acceptable wait times and age (the older the age, the shorter the acceptable wait times). It also revealed that participants who practiced at either an academic institution or a community practice expected shorter wait times than the survey respondents that practiced in both settings. There was a statistically significant correlation indicating that participants practicing in an academic center had longer acceptable wait times for surgery in MTC than those in a community practice.

Specifically for the question regarding a diagnosis of atypia on fine needle aspiration, a statistically significant correlation was found for acceptable wait times to surgery and those with head and neck oncology fellowships. Participants with a fellowship in head and neck oncology had comparably longer acceptable wait times to surgery than their colleagues. This significance was not present in the other proposed clinical scenarios. There were no other statistically significant findings within the linear regression model.

## Discussion

At the time of publication, this study is the first to examine thyroid surgery wait times. Our results from consensus data indicate a statistically significant difference between the actual and appropriate wait times for all thyroid surgical scenarios surveyed (except in the scenario of medullary thyroid cancer wait times). One of the possible explanations for inappropriately long wait times is because of the excellent prognosis associated with most types of thyroid cancer. Also, many patients with thyroid nodules do not have a diagnosis of cancer prior to surgery due to non-diagnostic FNAs. These factors contribute to a decreased sense of urgency when treating thyroid nodules and cancers.

We hypothesize that appropriate wait times for a diagnosis of medullary thyroid cancer do not differ significantly from actual wait times for several reasons. Firstly, medullary thyroid cancer has a low incidence (< 10% of all thyroid cancers) and therefore respondents likely have limited clinical experience to report with very low sample sizes [13]. Secondly, given it’s a more aggressive thyroid malignancy, a known diagnosis is likely treated more promptly than other thyroid malignancies [14]. The scarcity and severity of these cases justify prompt treatment.

Wait times for assessment of new consults also did not differ significantly between actual and appropriate. This finding may represent the fact that clinic time is more accessible than OR time. It appears participating physicians feel that they are triaging new consults appropriately and are able to assess these patients within an appropriate timeframe. We can therefore infer that the perceived bottleneck occurs when these patients are scheduled and waiting for surgery. This is not surprising given the widespread shortages in OR time, staffing, and resources.

The statistically significant negative linear regression between acceptable waiting times and a diagnosis of MTC at an academic institution (versus a community setting) could suggest that MTC, a more aggressive diagnosis, are more worrisome to those in the community setting than the academic institutions. This finding may be reflective of comfort level in managing a diagnosis of MTC; the larger centers are more likely to have increased experience with this pathology, which is possibly the cause of the difference in perceived urgency.

A similar negative regression was noted between wait times and age of surgeon. This finding could be reflective of the change in resource availability over the course of the older surgeons careers versus a differing of attitudes between generations. Finally survey participants who practice in either academic or community settings seem to expect shorter waiting times than those who practice in both; perhaps because those who practice in both settings have a better understanding of the resource limitations in each setting.

A study similar to ours was conducted on a much larger scale as part of the Canadian Pediatric Surgical Wait Time Project in 2011 [15,16]. This is an example of a national collaborative effort of a specialist group to address their wait times. Through workshops, specialists representing all pediatric subspecialties indentified all diagnoses they encounter, assigned a priority level to each diagnosis, and each priority level a corresponding target wait time [17]. The consensus-based targets resulting from this project have been adopted as provincial standards in two provinces thus far, promoting some continuity in wait times among different provincial jurisdictions. When compared against current wait time data, they can be used by practitioners, institutions, and government to make informed decisions regarding patient triage and management, resource allocation, and sharing of best practices [15]. This type of national effort can be used as an example for other areas of surgery, and in this case, for evaluating standardized thyroid surgery wait time targets.

The Wait Time Alliance (WTA) was formed by physicians in 2004 after the implementation of the *10 year plan to strengthen healthcare*. They have contributed to many wait time reduction efforts, including the development of benchmarks for the five priority areas identified in the plan [5]. Membership consists of multiple specialty societies across Canada, and recently expanded to include specialties beyond the initial five priority areas addressed in 2004. However, there is currently no representation for Otolaryngology- Head and Neck Surgery. Both the WTA and the government bodies are calling for membership and benchmarks across all specialty areas [6,17].

Our study has several limitations. Firstly, response rate could not be calculated due to our inability to measure precisely how many CSO-HNS members are performing thyroid surgery. There is also a physician population within Canada of non CSO-HNS members who perform thyroid surgery, such as general surgeons, who were not accounted for in this study. Secondly, our results have been generated by consensus, and are not necessarily evidence-based. However, it is acknowledged that there is a lack of evidence-based literature in the study of wait times, and specifically wait times for thyroid surgery. Conversely, physician consensus provides valuable clinical context in the development of wait time benchmarks, an element that is not addressed in evidence-based studies [5]. Wait time data can be difficult to interpret without considering the clinical context [15].

## Conclusions/recommendations

We feel that an increased accountability by the healthcare system will help improve the delivery of care. Accountability and dissemination of wait time data to the public was identified as a barrier to the progress of the 2004 health accord [18]. With this study, we hope to contribute to wait time reduction efforts in the field of Otolaryngology. With the number of thyroid cancer diagnoses and surgeries increasing [8,10], our hope is that this information will help to develop national wait time targets and subsequently improve delivery of care to this patient population.

The authors recommend that the CSO-HNS develop national thyroid surgery wait time targets for the various thyroid pathologies, including suspicious nodules with non-diagnostic FNAs. We also recommend that the CSO-HNS consider membership in the Wait Time Alliance, to demonstrate this specialty’s commitment to the reduction of wait times, and to make a meaningful contribution to this ongoing national effort.

## Competing interests

The authors declare that they have no competing interests.

## Authors’ contributions

MB, SMT, JT and RH conceptualized the study. MB was responsible for drafting the survey, assisting with survey distribution, data acquisition, analysis, and editing manuscripts. PM formatted the online survey and wrote and revised manuscripts. SM performed data collection and statistical analysis. All authors read and approved the final manuscript.
